# Alignment and photooxidation dynamics of a perylene diimide chromophore in lipid bilayers†

**DOI:** 10.1039/d2me00243d

**Published:** 2023-02-22

**Authors:** Novitasari Sinambela, Richard Jacobi, David Hernández-Castillo, Elisabeth Hofmeister, Nina Hagmeyer, Benjamin Dietzek-Ivanšić, Leticia González, Andrea Pannwitz

**Affiliations:** a Institute of Inorganic Chemistry I, Ulm University Albert-Einstein-Allee 11 89081 Ulm Germany andrea.pannwitz@uni-ulm.de; b Institute of Theoretical Chemistry, Faculty of Chemistry, University of Vienna Währinger Straße 17 1090 Vienna Austria leticia.gonzalez@univie.ac.at; c Doctoral School in Chemistry (DoSChem), University of Vienna Währinger Straße 42 1090 Vienna Austria; d Vienna Research Platform on Accelerating Photoreaction Discovery, University of Vienna Währinger Straße 17 1090 Vienna Austria; e Institute of Physical Chemistry and Abbe Center of Photonics, Friedrich Schiller University Jena Helmholtzweg 4 Jena 07743 Germany; f Leibniz Institute of Photonic Technology (IPHT), Research Department Functional Interfaces Albert-Einstein-Straβe 9 Jena 07745 Germany

## Abstract

We present a method of enabling photochemical reactions in water by using biomimetic, water-soluble liposomes and a specifically functionalized perylene diimide chromophore. Linking two flexible saturated C4-alkyl chains with terminal positively charged trimethylammonium groups to the rigid perylene diimide core yielded **[**1**]**^**2+**^ allowing for its co-assembly at the lipid bilayer interface of DOPG liposomes (DOPG = 1,2-dioleoyl-*sn-glycero*-3-phospho-(1′-*rac*-glycerol)) with a preferred orientation and in close proximity to the water interface. According to molecular dynamics simulations the chromophore aligns preferably parallel to the membrane surface which is supported by confocal microscopy. Irradiation experiments with visible light and in the presence of a negatively charged, water-soluble oxidant were slower in the DOPG-membrane than under acetonitrile–water reaction conditions. The generated radical species was characterized by EPR spectroscopy in an acetonitrile–water mixture and associated to the DOPG-membrane. Time-resolved emission studies revealed a static quenching process for the initial electron transfer from photoexcited **[**1**]**^**2+**^ to the water soluble oxidant. The findings presented in this study yield design principles for the functionalization of lipid bilayer membranes which will be relevant for the molecular engineering of artificial cellular organelles and nano-reactors based on biomimetic vesicles and membranes.

## Abstract 2

Enabling the photochemical redox process of organic chromophores in water.

## Graphical abstract

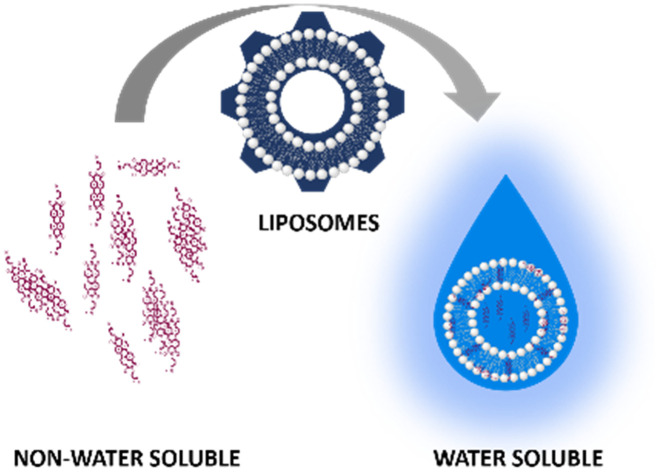

**Design, System, Application**

The system presented here demonstrates a design principle of how molecular species such as perylene diimides can be incorporated into lipid bilayer membranes in a preferred orientation and parallel to the membrane surface. Specifically, the functionalization of a rigid perylene diimide core with two flexible saturated C4-alkyl chains and terminal positively charged trimethylammonium groups leads to membrane anchorage on negatively charged membrane surfaces. This methodology is relevant for the design of molecular lipid bilayer hybrid materials and provides an underexplored opportunity to enable (photo-)chemical reactions with high spatial control which enables reaction confinement at the lipid bilayer membrane and for compartmentalization within the inner aqueous compartment of water-soluble vesicles. The findings presented here are relevant within the context of artificial cellular organelles and nano-reactor design.

## Introduction

In the context of solar light energy conversion including light-driven water splitting to form oxygen and hydrogen, as well as enzymatic catalysis, photoredox catalysis and water detoxification, it is oftentimes essential to be able to use water as solvent or co-solvent. However, water solubility for a given light active molecule or material typically requires synthetic efforts or adapted material or molecular systems design strategies.^1–3^

In nature, the challenge of chemical reactions in water with partly or fully water insoluble reaction partners is solved by the compartmentalization of reaction spaces using lipid bilayer membranes which span cells, vesicles and cellular compartments. Especially interesting for light energy conversion are the processes in natural photosynthesis, where solar energy conversion is enabled within hydrophobic membranes and their interface towards aqueous compartments. The most essential photochemical reaction step in photosynthesis is light-driven charge transfer from the photoexcited light-absorbing molecule towards an electron acceptor or an electron donor. An important aspect here is the precise orientation of the chromophores within the membrane.^4^

By mimicking nature, we are here exploring a method for photochemical redox processes to be performed in water and at the interface of biomimetic phospholipid bilayers using an organic, molecular chromophore embedded with the membrane and at the interface with water. In this case the lipid bilayer membrane builds up vesicles which can be varied in size and shape^5^ and can prospectively act as nano-photoreactors.^6^ Successful light-driven CO_2_ reduction, hydrogen evolution, water oxidation and NADH oxidation at biomimetic lipid bilayer vesicles (liposomes) have previously been reported using metal based photosensitizers based on ruthenium polypyridyl complexes.^5–12^ Our motivation to investigate an organic chromophore is to enable light-driven chemical transformations with photosensitizers from more abundant resources. By constructing a photoactive molecular system with lipid bilayers, the microenvironment of the individual chromophore is altered from a typical solution environment which can change and oftentimes improves the reaction dynamics or chromophore stability.^5,11^ We therefore characterized the light driven oxidation dynamics between an organic chromophore and a water soluble oxidant.

**Fig. 1:**
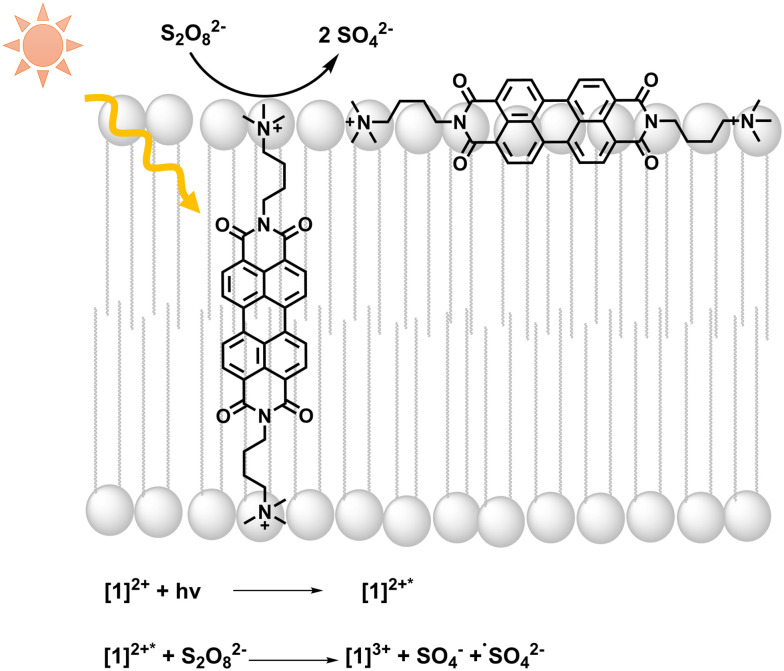
Light absorption by **[**1**]**^**2+**^ is followed by photooxidation of **[**1**]**^**2+**^ by persulfate; equations of initial light-driven reactions are included.

The organic chromophore investigated here is a substituted version of perylene diimide (PDI). PDIs are known for their activity as photosensitizers in photoredox catalysis in organic solution and their aggregation behavior in solution.^13–16^ Typically, PDIs absorb light in the visible region of the solar spectrum, and therefore they enable low-energy photon emission compared to UV light which might be relevant in the technological context using LED light sources for photochemical reactions. In this study, PDI has been substituted with two butyl trimethyl ammonium alkyl chains at the imine-nitrogens, forming *N*,*N*′-di(butylenetrimethylammonium)-3,4,9,10-perylenediimide **[**1**]**^**2+**^ as PF_6_ salt (**[**1**]**^**2+**^, see Fig. 1). In principle, the length of the molecule matches the thickness of the phospholipid bilayers of around 2.4 nm, potentially enabling preferred orientations at artificial lipid bilayers, such as 1,2-dioleoyl-*sn-glycero*-3-phospho-(1′-*rac*-glycerol) (DOPG) bilayers.^5,17,18^ Here, the specific orientation of **[**1**]**^**2+**^ within the lipid bilayer was investigated explicitly using all-atom molecular dynamics (MD) simulations. Light-driven electron transfer to an oxidant in solution generates a PDI based radical which was studied by EPR spectroscopy in homogeneous solution and at DOPG liposomes, while the detailed difference of the reaction dynamics of the homogeneous solution and at DOPG liposomes was resolved using time resolved fluorescence spectroscopy, giving insights into the reaction dynamics at the biomimetic membrane–water interface.

## Results

### Synthesis of **[**1**]**^**2+**^ and lipid bilayer integration

The substituted PDI **[**1**]**^**2+**^ was synthesized from perylene-3,4,9,10-tetracarboxylic dianhydride as described in the ESI:† a condensation reaction with (4-aminobutyl) dimethylamine yielded an uncharged PDI which was then methylated with CH_3_I to introduce charged trimethyl amine groups. After salt exchange using an aqueous solution of NH_4_PF_6_, the PF_6_-salt of **[**1**]**^**2+**^ was isolated with an overall yield of 76% over two chemical reactions and one salt exchange.

**Fig. 2:**
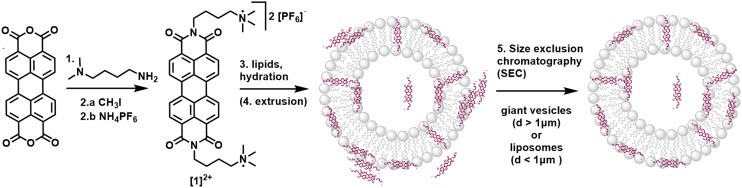
Synthesis of **[**1**]**^**2+**^ starting from perylene-3,4,9,10-tetracarboxylic dianhydride and incorporation of **[**1**]**^**2+**^ into vesicle membranes. Reaction conditions and yields: 1.) 4 eq. (4-aminobutyl)dimethylamine, i-butanol, 90 °C, 24 h, 95%; 2.a) 3 eq. CH_3_I, toluene, reflux, 3 h; 2.b) excess NH_4_PF_6_, H_2_O, 65 °C, 76%; 3.) phosphate buffer, 100 : 1 : 1 (*n*/*n*/*n*) DOPG lipids/14 : 0 PEG2000 PE/ **[**1**]**^**2+**^; 4.) extrusion; 5.) size exclusion chromatography (SEC).

Integration of **[**1**]**^**2+**^ into liposomes was only possible when using the negatively charged 1,2-dioleoyl-*sn-glycero*-3-phospho-*rac*-(1-glycerol) sodium salt (DOPG) as the phospholipid. Neutrally charged lipids such as DMPC (1,2-dimyristoyl-*sn-glycero*-3-phosphocholine) did not take up **[**1**]**^**2+**^, indicating a benefit of the electrostatic attraction between **[**1**]**^**2+**^ and the lipid. For liposome preparation, DOPG as the main lipid, **[**1**]**^**2+**^ and the PEGylated lipid 1,2-dimyristoyl-*sn-glycero*-3-phosphoethanolamine-*N*-[methoxy(polyethylene glycol)-2000] (ammonium salt) (14 : 0 PEG2000 PE) for steric stabilization of the vesicles were combined in the desired molar ratio in a chloroform–acetonitrile mixture (here: 100 : 1 : 1). Solvent evaporation yielded a lipid film which was hydrated with an aqueous buffer to yield giant vesicles for confocal microscopy, and additional extrusion yielded small vesicles (liposomes) with typical hydrodynamic diameters of 110–120 nm indicating stability of vesicles containing **[**1**]**^**2+**^. In some cases, size exclusion chromatography was performed additionally to remove **[**1**]**^**2+**^ outside of the liposomes (Fig. 2). More details on the vesicle preparation are described in the experimental part.

### Spectroscopic characterization

**Fig. 3:**
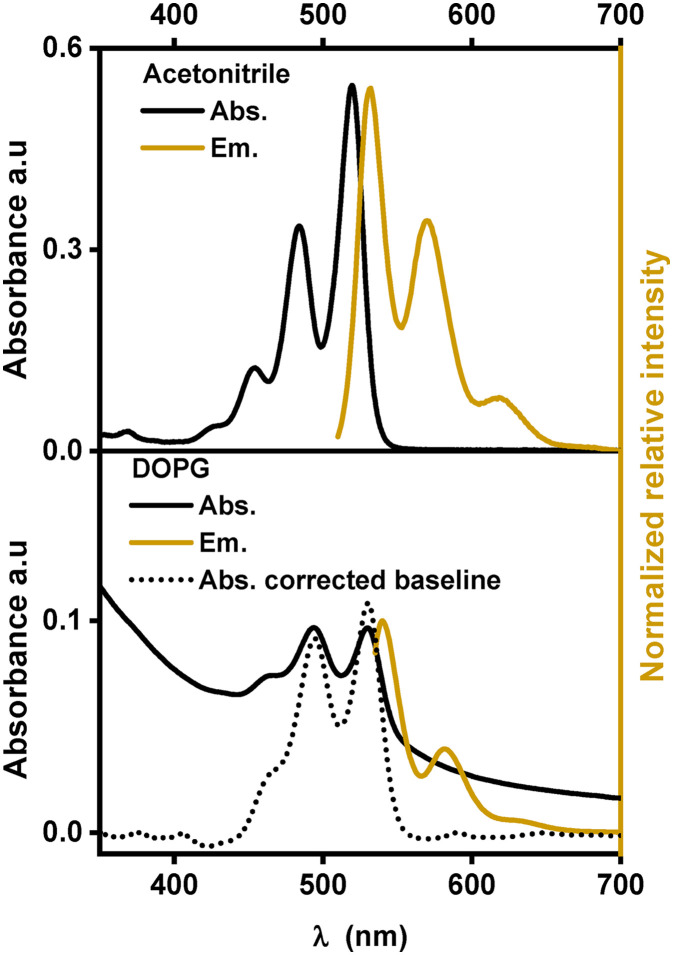
Absorption and emission spectra of **[**1**]**^**2+**^ in various environments.

Similar to other substituted PDIs, **[**1**]**^**2+**^ is soluble in organic solvents and strongly absorbs visible light in the region between 400 nm and 550 nm with three characteristic absorption bands, indicating the dominance of monomeric species of **[**1**]**^**2+**^ over aggregation.^14,19^ In acetonitrile, these absorption bands are at 453, 484 and 520 nm with extinction coefficients ranging between 14 000 and 61 000 M^−1^ cm^−1^. In a 1 : 1 (V : V) mixture of acetonitrile and water, the absorption peaks are shifted to lower energy by 7 to 10 nm indicating an impact of the solvent environment on the electronic levels of the chromophore. Such bathochromic shifts in the PDI spectra were previously observed with protic solvents and are probably due to stabilization of the more polarizable excited states upon hydrogen bonding with the solvent and the formation of J-aggregates.^13,20–22^ In the emission spectra, three maxima and a similar red-shift were observed when going from acetonitrile (531, 571 and 620 nm) to the acetonitrile/water 1 : 1 (V : V) mixture (540, 580 and 630 nm) (see Fig. 3). The fluorescence lifetime determination yielded mono-exponential decays in acetonitrile and in acetonitrile/water 1 : 1 (V : V) with a lifetime of (4.1 ± 0.1) ns or (4.2 ± 0.1) ns, respectively (see Table 1). In other PDI-based chromophores, similar fluorescence lifetimes of around 4 ns were reported.^23–25^

**Table 1:** Spectroscopic data for **[**1**]**^**2+**^

Solvent or lipid	*λ*_Abs_ (nm) (*ε* [M^−1^ cm^−1^])	*λ*_em_ (nm)	*τ* (ns)
Acetonitrile	453 (14000), 484 (38000), 520 (61000)	531, 571, 620	4.1 ± 0.1
Acetonitrile/water (1 : 1)	461(18700), 490 (49000), 527 (76000)	540, 580, 630	4.2 ± 0.1
DOPG liposomes^a^	460, 494, 530	540, 582, 630	4.1 ± 0.1
DOPG liposomes^b^	460, 494, 530	540, 582, 630	4.0 ± 0.1

a DOPG : (14 : 0 PEG2000 PE) : **[**1**]**^**2+**^ = 100 : 1 : 1; in phosphate buffer (10 mM, pH 7.0).

b DOPG : (14 : 0 PEG2000 PE) : **[**1**]**^**2+**^ = 100 : 1 : 1; in phosphate buffer (10 mM, pH 7.0) after size exclusion.

Fig. 3 shows the absorption and emission spectra of **[**1**]**^**2+**^ in DOPG-liposomes. Upon manual baseline correction of the Tyndall scattering in the absorption spectrum (the dotted line in Fig. 3), the similarity between the absorption spectra in solution and in liposomes becomes evident. In DOPG liposomes the three absorption and emission bands of **[**1**]**^**2+**^ are at similar wavelengths to those in acetonitrile/water 1 : 1 (V : V); however, the vibrational bands at 460 nm and 640 nm respectively appear as a shoulder instead of distinct peaks, indicating some level of aggregation within the lipid bilayers. Typically, the assembling chromophores within hydrophobic lipid bilayers would yield hypsochromic spectral shifts, as the more hydrophobic environment typically destabilizes the more polar or more polarizable excited states.^26^ However, the incorporation into lipid membranes shows a bathochromic shift with respect to the organic solvent and only a minor shift by one to two nanometers compared to acetonitrile/water 1 : 1 (V : V), which indicates that the chromophore is exposed to hydrogen bonding water molecules when embedded within the lipid bilayer. In terms of fluorescence lifetime, embedding **[**1**]**^**2+**^ into the lipid bilayer of DOPG liposomes slightly reduced the excited state lifetime to around 4.0 ns.

**Fig. 4:**
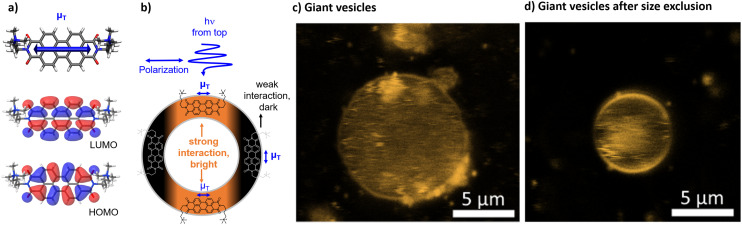
a) DFT optimized structures of **[**1**]**^**2+**^ including the shape of the HOMO and LUMO and the directionality of the calculated transition dipole moment (*μ*_T_). b) Illustration of the interactions of a polarized light source with the transition dipole moment of **[**1**]**^**2+**^ in the lipid bilayer. c and d) Confocal images of giant vesicles with DOPG : (14 : 0 PEG2000 PE) : **[**1**]**^**2+**^ = 100 : 1 : 1; in phosphate buffer (10 mM, pH 7.0) upon fixation in an agarose hydrogel d) without size exclusion chromatography and e) after size exclusion chromatography.

Size exclusion chromatography after vesicle preparation typically removes water soluble and smaller aggregates from the bulk solution around the vesicles. Doing so here had no effect on the spectral data, but confocal microscopy of giant vesicles showed that, clearly, some aggregates could be removed from the bulk around the vesicles (Fig. 4c and d).

### Confocal microscopy and the preferred orientation

The integration into the lipid bilayer was proven by confocal fluorescence microscopy as depicted in Fig. 4c and d showing typical giant vesicles loaded with around 1 mol% **[**1**]**^**2+**^. In these confocal microscopy images, the sample is irradiated with a polarized laser source at 488 nm and the microscopy image is collected in the range of emission of **[**1**]**^**2+**^, at 503–750 nm. As seen in Fig. 4c and d, the shape of a round vesicle is emissive, which indicates targeted incorporation of the fluorescent **[**1**]**^**2+**^ into the lipid bilayer of these vesicles. It was observed that size exclusion chromatography could remove some aggregated **[**1**]**^**2+**^ from the bulk around the giant vesicle but that some aggregates have been enclosed within the vesicle. The latter is shown by the fluorescent matter in the inner volume of the vesicle in Fig. 4c and d. As the giant vesicles are fixated within the agarose hydrogel, the vesicle itself can be resolved well, but the inner aqueous compartment of the vesicle contains a highly mobile aqueous buffer, so that the fluorescent matter in the inner compartment cannot be resolved well with a microscope and appears to be rather diffuse. Furthermore, a double half-moon effect is seen within this image, where the equatorial region of the vesicle remains dark while the north and south poles are brightly emissive. This double half-moon effect is typical for chromophores with one main transition dipole moment at the excitation wavelength and a preferred orientation within the relatively rigid matrix of the lipid bilayer.^18,26^ As revealed by time-dependent density functional theory (TD-DFT), the transition of **[**1**]**^**2+**^ associated with emission, *i.e.*, between the first excited singlet state and the ground state, is of ππ*-character, as indicated by the HOMO and LUMO (see Fig. 4a). The corresponding transition dipole moment oscillates along the main molecular axis between the two imine-nitrogens (see Fig. 4b) which enables orientation dependent interaction with the polarized incident laser light source and selective excitation (and emission) of the well-aligned chromophores with respect to the incident polarized laser. As schematized in Fig. 4b, the alignment of the molecules of **[**1**]**^**2+**^ parallel to the membrane surface leads to the observed half-moon effect as this requires a preferred orientation of **[**1**]**^**2+**^ with respect to the membrane surface. This would enable easy access of water-soluble reactants to the chromophore **[**1**]**^**2+**^ which is therefore close to the membrane–water interface. However, in previous work, luminescent chromophores with rigid backbones in a vesicle membrane showed the double halfmoon effect as well but most likely due to a preference for a transmembrane alignment.^18,26^ Using MD simulation, we have elucidated the orientation preference of **[**1**]**^**2+**^ within the lipid double layer.

### MD simulations and orientation

**Fig. 5:**
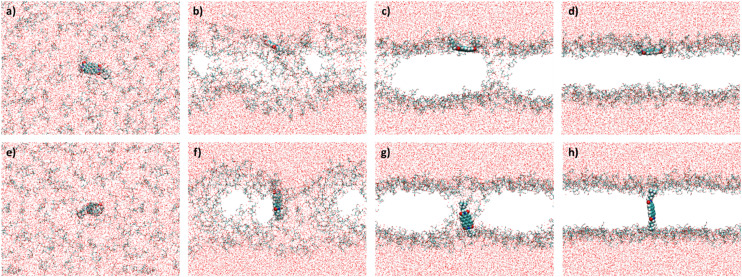
Stages of the membrane assembly in the simulations. a–d) Parallel insertion of **[**1**]**^**2+**^ (62–81%, see Table 3); e–h) transmembrane insertion of **[**1**]**^**2+**^ (19–38%, see Table 3). Color code: red = oxygen, white = hydrogen, blue = nitrogen, yellow = phosphorus, and turquois = carbon. The alkyl tails of the lipid bilayer are omitted for clarity.

We performed 50 MD simulations in total, where **[**1**]**^**2+**^ was put into a cubic box of 90 Å side length. In 20 of these 50 trajectories, 150 DOPG molecules were randomly distributed in an aqueous solution alongside **[**1**]**^**2+**^. In the remaining 30 trajectories, 170 DOPG molecules were placed into the box to simulate the assembly of the lipid bilayer and the alignment of **[**1**]**^**2+**^ within it. Two different lipid concentrations were used to investigate the influence of lipid concentration on the outcome; as in the experimental preparation of the giant vesicles and liposomes, the solvent was evaporated and therefore the lipid concentration was not constant. The stages, which the MD simulations underwent, were similar throughout all the trajectories and have been previously reported for the self-assembly of lipid bilayer membranes.^27,28^ These are exemplarily represented in Fig. 5 for **[**1**]**^**2+**^ that inserts parallel to the assembled membrane (Fig. 5a–d) and for one inserting transmembrane (e–h). At first, all molecular components are randomly distributed in the simulation box (a and e). Usually within the first 10 ns, the phospholipids aggregate, with **[**1**]**^**2+**^ bound to the aggregates (b and f). The aggregates assemble in one plane, forming a membrane, which is penetrated by water pores (c and g). As is apparent from our simulations, the configuration of **[**1**]**^**2+**^ relative to the water pores is impacting the final insertion. In the final stage, the water pores disappear and a fully assembled bilayer is formed. This vanishing of the pores can take a couple of hundred of nanoseconds, especially in the trajectories with a higher number of lipid molecules. If **[**1**]**^**2+**^ is in the pore when the water molecules leave the hydrophobic area of the membrane, **[**1**]**^**2+**^ assembles the transmembrane. This does not happen if **[**1**]**^**2+**^ does not penetrate through one of the pores. As there appears to be some chance of whether **[**1**]**^**2+**^ penetrates a pore when the pore disappears, there is also a statistical distribution between parallel and transmembrane insertions over the trajectories. The results of the assembly simulations are presented in Table 2.

**Table 2:** Results of the assembly simulations of **[**1**]**^**2+**^

DOPG molecules	Trajectories with **[**1**]**^**2+**^ assembled in the membrane	Parallel insertions	Transmembrane insertions
150	16	13 (81%)	3 (19%)
170	21	13 (62%)	8 (38%)

The MD simulations predict a preference for the parallel insertion mode of **[**1**]**^**2+**^ into the membrane. This trend is particularly clear from the simulations with 150 DOPG molecules with *ca.* 80% of the trajectories displaying parallel insertions (Fig. 5a–d) but also evident in the simulations using more DOPG molecules, with the parallel insertion mode still amounting to over 60% of the completed trajectories. We therefore conclude that the parallel **[**1**]**^**2+**^ should be the dominant species responsible for the double half-moon effect exhibited in the confocal microscopy images. However, an important finding of the simulations is that in both sets there are non-negligible amounts of transmembrane **[**1**]**^**2+**^ (Fig. 5e–h), indicating that both insertion modes occur in the membrane, and any subsequent analysis cannot be limited to a single orientation. In both orientations, **[**1**]**^**2+**^ is partially exposed to hydrogen bonding water molecules from the aqueous phase and can potentially engage in light-driven oxidation reactions with water soluble reactants.

### Photooxidation

To see if the membrane-associated **[**1**]**^**2+**^ could engage in light-driven reactions with reactants in the aqueous bulk, the respective DOPG liposomes were exposed to the water-soluble oxidant sodium peroxydisulfate (Na_2_S_2_O_8_) and a 470 nm light source. To exclude effects of vesicle size and surface curvature on the reaction dynamics,^6,29^ unilamellar, monodisperse vesicles with an average hydrodynamic diameter of around 120 nm were prepared, providing a high surface area compared to giant multilamellar vesicles. The reaction progress was monitored using time resolved and steady state UV-vis absorption spectroscopy and emission spectroscopy and the radical generation was additionally characterized using electron paramagnetic resonance spectroscopy (EPR).

**Fig. 6:**
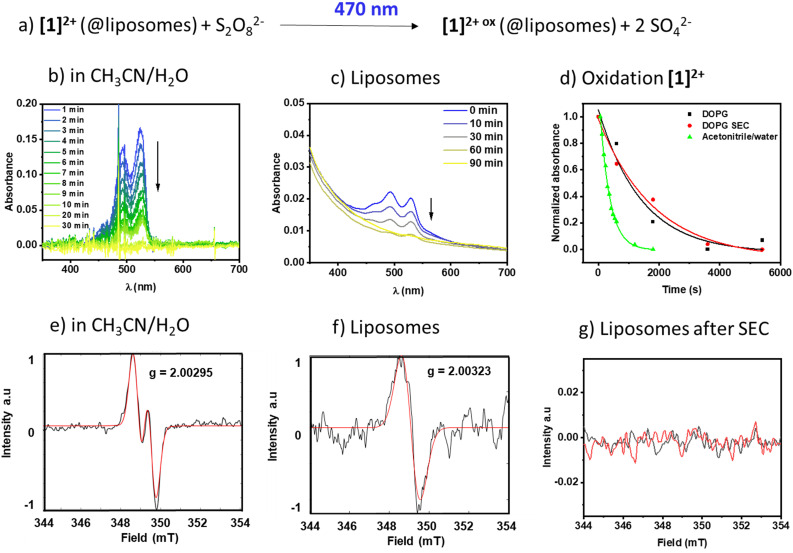
a) Photooxidation of **[**1**]**^**2+**^ and temporally resolved UV-vis spectra in b) acetonitrile/water 1 : 1 (V : V) and c) DOPG : (14 : 0 PEG2000 PE) : **[**1**]**^**2+**^ = 100 : 1 : 1 liposomes in phosphate buffer (10 mM, pH 7.0) after size exclusion. d) Temporal evolution of the PDI absorbance at 527 nm (acetonitrile/water) and 530 nm (DOPG) in different environments. e–g) EPR spectra of the photooxidized PDI after 18 h irradiation at 455 nm in e) acetonitrile/water and f and g) liposomes with DOPG : (14 : 0 PEG2000 PE) : **[**1**]**^**2+**^ = 100 : 1 : 1 in phosphate buffer (10 mM, pH 7.0) f) without size exclusion and g) after chromatography (SEC).

In pure water **[**1**]**^**2+**^ was insufficiently soluble and therefore we added acetonitrile to generate a 1 : 1 (V : V) mixture of acetonitrile and water to solubilize both **[**1**]**^**2+**^ and the oxidant sodium persulfate in a homogenous solution. Fig. 6b shows the respective time-dependent spectra under homogeneous conditions, and Fig. 6d shows the absorbance *vs.* time profiles, demonstrating a fast consumption of **[**1**]**^**2+**^ with a rate constant of (3.04 ± 0.1) 10^−3^ s^−1^. The photogenerated radical **[**1**]**^**3+**^**˙** was characterized under comparable homogeneous conditions using EPR (Fig. 6e) and shows a signal with *A*_1_ = 59 MHz and *A*_2_ = 7 MHz and a *g*-factor of *g* = 2.00295 compatible with a radical centered on one carbon and one hydrogen, which is in agreement with the formation of *J*-aggregates of **[**1**]**^**2+**^ in aqueous solution.

Upon incorporation of **[**1**]**^**2+**^ into the membrane of DOPG liposomes, the rate of oxidation becomes slower, as seen in the time-dependent evolution of the spectra in Fig. 6c and in 6d. Furthermore, the kinetics of the photooxidation in liposomes do not depend on the purification of the liposomes ((0.70 ± 0.3) 10^−3^ s^−1^*vs.* (0.55 ± 0.1) 10^−3^ s^−1^ without or with size exclusion chromatography purification, respectively). However, the EPR signal of only the non-size excluded liposomes could be detected under the measurement conditions applied here. It shows a singlet at *g* = 2.00323, compatible with an unpaired electron localized on a carbon. It is expected that upon inclusion of a radical into a solid-state matrix, it is difficult for the radicalized molecule to align within the magnetic field of the resonator, resulting in line broadening due to anisotropic effects. As a radical signal is visible in the non-size excluded samples, we assume that there are small amounts of mobile **[**1**]**^**2+**^ molecules that can rotate and align to some extent in the magnetic field and this appears as a broad signal in the EPR spectrum. Size exclusion seems to remove this fraction of mobile molecules and thereby removes the signature of a radical at the applied resolution, which might be due to strong band broadening due to the solid-like state matrix of the lipid bilayer.^30^ Removal of **[**1**]**^**2+**^ from the bulk aqueous solution *via* size exclusion is supported by a diminished absorption of the PDI signal at 527 nm (see Fig. 6c*vs.* Fig. S2.1†). Based on the extinction coefficient in acetonitrile/water and neglecting the scattering effect we estimate that the overall concentration of **[**1**]**^**2+**^ in the sample is reduced from around 1.3 μM to around 0.3 μM, which indicates that a large portion of the chromophore in solution could be removed. As the UV-vis spectroscopic signatures and conversion rates in liposomes are practically identical, we conclude that the radical species oxidized **[**1**]**^**2+**^ is formed nevertheless.

**Fig. 7:**
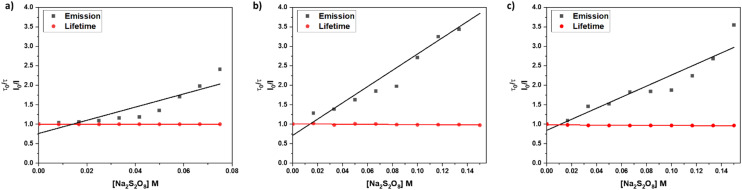
Stern–Volmer quenching experiments for different systems: a) acetonitrile/water 1 : 1 (V : V), b) DOPG : (14 : 0 PEG2000 PE) : **[**1**]**^**2+**^ = 100 : 1 : 1 liposomes in phosphate buffer (10 mM, pH 7.0) without size exclusion chromatography, c) DOPG : (14 : 0 PEG2000 PE) : **[**1**]**^**2+**^ = 100 : 1 : 1 liposomes in phosphate buffer (10 mM, pH 7.0) after size exclusion chromatography.

In an emission quenching assay, the so-called Stern–Volmer plot, we observed that the photoexcited state of **[**1**]**^**2+**^ is quenched at various concentrations of the oxidant Na_2_S_2_O_8_. Quenching of homogeneously dissolved **[**1**]**^**2+**^ in 1 : 1 (V : V) acetonitrile–water mixture occurred with a Stern–Volmer constant with different values for the quenching of the emission spectrum's intensity and lifetime with *K*_SV_(*I*_0_/*I*) = 17.0 ± 2.9 and *K*_SV_(*τ*_0_/*τ*) = 0.04 ± 0.02, respectively, which indicates a static quenching process as the intensity-based Stern–Volmer constant is positive and the lifetime-based Stern–Volmer constant is close to zero.^7,31,32^ Upon incorporation of **[**1**]**^**2+**^ into liposomes the intensity and lifetime-based Stern-Volmer constants differ from each other with *K*_SV_(*I*_0_/*I*) = 20.9 ± 1.9 and *K*_SV_(*τ*_0_/*τ*) = −0.17 ± 0.02, respectively. The lifetime-based Stern–Volmer constant appears to be slightly negative, however, when comparing the actual lifetimes, no significant effect is observed, as the lifetimes stay constant within the experimental error of the lifetime measurements. Comparing it to the intensity-based Stern–Volmer constant, it is close to zero, indicating static quenching. Within liposomes after size exclusion, the intensity-based lifetime Stern–Volmer constant is *K*_SV_(*I*_0_/*I*) = 14.2 ± 1.9 while the lifetime-based Stern–Volmer constant approaches zero *K*_SV_(*τ*_0_/*τ*) = −0.17 ± 0.02 which strongly indicates a static quenching process (Fig. 7).^7,31,32^ Such static quenching processes are typical for singlet emitters with short excited state lifetimes where a pre-associated chromophore–quencher adduct needs to be formed for the quenching process to take place (Table 3).^31^

**Table 3:** Oxidation rate constants, Stern–Volmer quenching constants and quenching constants of **[**1**]**^**2+**^ in acetonitrile/water and DOPG membranes, monitored at the absorption maximum

Solvent or lipid	*k*_ox_ (10^−3^ s^−1^)	*K*_SV_ from *I*_0_/*I* (M^−1^)	*K*_SV_ from *τ*_0_/*τ* (M^−1^)	*k*_q_ (10^−9^ M^−1^ s^−1^) from *I*_0_/*I*
Acetonitrile/water	3.04 ± 0.1	17.0 ± 2.9	0.04 ± 0.02	70.9 ± 0.03
DOPG liposomes^a^	0.70 ± 0.3	20.9 ± 1.9	−0.17 ± 0.02	85.9 ± 0.02
DOPG liposomes^a^^,^^b^	0.55 ± 0.1	14.2 ± 1.9	−0.17 ± 0.02	56.8 ± 0.04

a DOPG : (14 : 0 PEG2000 PE) : **[**1**]**^**2+**^ = 100 : 1 : 1; in phosphate buffer (10 mM, pH 7.0).

b After size exclusion.

**Fig. 8:**
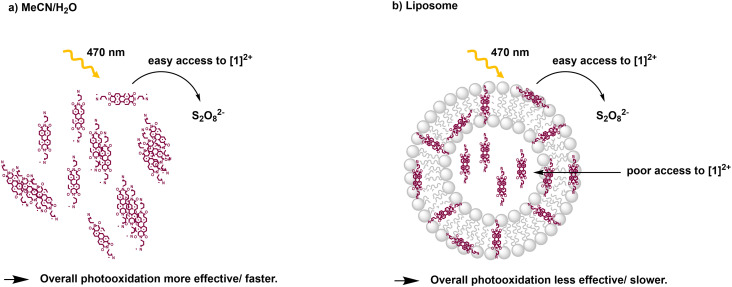
Illustration of photooxidation experiments for different systems: a) acetonitrile/water 1 : 1 (V:V); b) DOPG : (14 : 0 PEG2000 PE) : **[**1**]**^**2+**^ = 100 : 1 : 1 liposomes in phosphate buffer (10 mM, pH 7.0).

From the Stern–Volmer constants and the lifetime of the chromophore in the absence of a quencher, the quenching constant *k*_q_ can be calculated as follows:^31,33^*k*_q_ = *K*_SV_·*τ*_0_. Interestingly, all quenching constants *k*_q_ are very similar for all the investigated environments of **[**1**]**^**2+**^, however, the rate of photooxidation to complete oxidation of **[**1**]**^**2+**^ differs by around a factor of two between the homogeneous solution and liposomes (Table 3). We propose that these differences in the kinetics are due to the differences in the diffusion behavior of the water-soluble persulfate anions through the membrane, which access the chromophores at the inner membrane surface and aqueous compartment more slowly than in the homogenous environment (Fig. 8). This explanation is in line with reported transmembrane diffusion coefficients for ionic species of around 10^−3^ to 10^−8^ cm s^−1^ and reported cases of transmembrane diffusion of smaller ions.^34–36^

## Conclusions and outlook

In summary, **[**1**]**^**2+**^ forms *J*-aggregates in the presence of water and can be incorporated into negatively charged lipid bilayers of DOPG. According to results obtained from MD simulations, it preferably aligns parallel to the membrane surface although transmembrane orientations are also possible to a minor extent. Light-driven oxidation by the water-soluble, negatively charged oxidant persulfate takes place *via* a static quenching mechanism both in liposomes and in homogeneous solution due to electrostatic pre-association of both reactants. Despite similar excited state quenching dynamics, the photooxidation of **[**1**]**^**2+**^ is two factors faster in homogeneous solution compared to the lipid bilayer environment, which is attributed to the relatively slow diffusion of the oxidant through the lipid bilayer membrane to photooxidize the chromophore within the aqueous interior of the liposomes. These findings will be important for the design and engineering of molecular systems in the context of light-driven catalysis involving amphiphilic membrane materials and abundant organic photosensitizers.

## Experimental and methods part

All reagents were commercially available. Perylene tetracarboxylic dianhydride and methyl iodide were purchased from Merck. (4-Aminobutyl)dimethylamine was purchased from Enamine. The synthesis of **[**1**]**^**2+**^ was adapted from a previously reported procedure (ESI,† S1). All synthesis reactions were carried out under an inert atmosphere using standard Schlenk-techniques and argon gas. A Bruker DRX 400 spectrometer was used to obtain nuclear magnetic resonance (NMR) spectra recorded at 298 K. MestReNova was used for the evaluation of the spectra. The mass spectra of the compounds were obtained using a matrix assisted laser desorption ionization (MALDI) Bruker Reflex III mass spectrometer. *trans*-2-[3-(4-*tert*-Butylphenyl)-2-methyl-2-pro-penylidene]malononitrile was used as the matrix. Elemental analysis was performed by Mikroanalytisches Laboratorium Kolbe in Oberhausen, Germany. The elemental content of the molecules was reported as the element's mass fraction percentage. Absorption spectra were recorded on a JASCO V760 spectrometer or an Avantes AvaSpec. Starna fluorescence quartz cuvettes with a path length of 1 cm were used. Luminescence was recorded using a Horiba Jobin Yvon FluoroMax4 Plus C spectrophotometer equipped with a 150 W xenon lamp. The error of the lifetime was estimated to be 0.1 ns. The errors given in Table 3 are based on the error derived from the data fitting. In the case of the *K*_SV_ constants from *τ*_0_/*τ*, the slope value was very small, and we report the standard deviation of the slope fitting or 10% if the standard deviation was smaller than 10%. The size distribution of the hydrodynamic diameter (*Z*_Avg_) was measured at 20 °C with a Zetasizer Pro from Malvern operating at 633 nm with a scattering angle of 173°. Confocal microscopy images were obtained using a Leica TCS SP8 confocal microscope equipped with a 488 nm laser as the excitation source and detected in the range of 503–750 nm. Images were processed with LasX. Luminescence lifetimes were recorded with a DeltaPro system from Horiba Scientific using a 451 nm pulsed laser source and a 495 nm long pass filter; the instrument response function was measured with LUDOX silica nanoparticles.

### Preparation and characterization of giant vesicles and liposomes

Giant vesicles and liposomes were prepared as follows. The lipids 1,2-dioleoyl-*sn-glycero*-3-phospho-(1′-*rac*-glycerol) (DOPG) and 1,2-dimyristoyl-*sn-glycero*-3-phosphoethanolamine-*N*-[methoxy(polyethylene glycol)-2000] (ammonium salt) (14 : 0 PEG2000 PE) were prepared as stock solutions in CHCl_3_. To prepare a lipid film in a 5 mL round-bottom flask, a 1 mL stock solution of DOPG (5.0 mM) and 1 mL 14 : 0 PEG2000 PE (0.05 mM) in CHCl_3_ and 1 mL **[**1**]**^**2+**^ (0.05) in acetonitrile were combined. The organic solvents were evaporated under vacuum leading to deposition of the lipid on the flask wall. The film was dried under high vacuum for at least one hour and hydrated with 10 mM buffer phosphate (KH_2_PO_4_ (626 mg, 4.6 mmol), K_2_HPO_4_·3H_2_O (1.186 g, 5.2 mmol), and K_2_SO_4_ (1.70 g, 9.74 mmol) in Milli-Q water (1 L) to reach a final pH of 7.0). The dispersed lipid film was repeatedly freeze-thawed using liquid N_2_ and a water bath at room temperature yielding giant vesicles. To obtain liposomes of uniform size, the dispersion was extruded at room temperature through 200 nm cellulose membrane filters 11 times with an Avanti Polar Lipids mini-extruder. If applicable, the liposome mixture was subjected to a Sephadex G-25 size exclusion chromatography (SEC) column (6 cm length, 2 cm diameter) using phosphate buffer pH 7.0 as the eluent. Dynamic light scattering (DLS) on liposomes with and without SEC typically yielded a *Z*_Avg_-diameter of around 110–120 nm and a PDI of around 0.1. Confocal microscopy with a Leica TCS SP8 and sample wells (μ-Slide 8 Well ibiTreat) was used to characterize giant vesicles. Prior to microscopy, and if applicable, giant vesicles were subjected to SEC as described above. 100 μL of the giant vesicle sample was loaded to a well, and then 200 μl of a freshly prepared, air-cooled agarose solution (1 weight% in water) was added and mixed. Confocal microscopy measurements were conducted after the mixed solutions had settled at room temperature.

### Photoirradiation

The typical reactions were carried out with 5 μM of **[**1**]**^**2+**^ and 50 mM sodium persulfate mixed in a quartz cuvette with a total volume of 3 mL. The mixtures were stirred at room temperature and illuminated with a LED-stick (Nichia Corporation; NSPB500AS) (*λ* = 470 nm, 27 mW measured with a Hioki 3664 optical power meter set to 470 nm) which consisted of a custom-made reactor equipped with four ventilators to exclude heating of the samples.^37^ The samples were monitored *via* UV-vis spectroscopy after 0, 10, 30, 60, and 90 minutes.

### EPR measurements

Samples with a concentration of 10–4 mM **[**1**]**^**2+**^ and 50 eq. of Na_2_S_2_O_8_ in acetonitrile/water (V : V = 1 : 1) were prepared. For the measurements of **[**1**]**^**2+**^ integrated into DOPG liposomes, samples with a concentration of 10–4 mM **[**1**]**^**2+**^ and 10 mM DOPG and 50 eq. of Na_2_S_2_O_8_ were prepared in phosphate buffer pH 7.0. All sample solutions were transferred into a flat cell with a volume of approximately 200 μL under ambient conditions. CW EPR spectroscopic measurements were carried out at the X-Band using a Bruker ELEXSYS E500 spectrometer equipped with an SHQE resonator. Measurements were performed at room temperature with microwave powers of 5–6 mW and modulation amplitudes of 0.1 mT (without liposomes), 0.5 mT (with liposomes) and 0.3 mT (with liposomes SEC). A fiber-coupled LED (455 nm, Thorlabs M455F3) was installed for continuous irradiation of the samples in the resonator for up to 20 h. Simulation of experimental data was carried out with EasySpin.^38^

### Computational details

The transition dipole moment (*μ*_T_) of **[**1**]**^**2+**^ was computed using time-dependent density functional theory (TD-DFT) at the RB3LYP/def2-SVP level of theory,^39–42^ as implemented in the program package Gaussian16, revision C.01,^43^ using the optimized first excited singlet structure. Solvent effects for acetonitrile were included implicitly using the polarizable conductor-like calculation model (CPCM) by placing the solute in a cavity within the solvent reaction field.^44,45^ Dispersion interaction effects were corrected empirically using the Grimme D3 model with Becke–Johnson damping.^46,47^

Classical force field MD simulations of the lipid bilayer assembly were carried out to elucidate the orientation and alignment of **[**1**]**^**2+**^. To this aim, we randomly distributed 150 and 170 DOPG lipids (20 and 30 trajectories, respectively) and one molecule of **[**1**]**^**2+**^ in a cubic box of 90 Å side length. The remaining box volume was then filled with water molecules and KCl ions to ensure charge neutrality and attain a salt concentration of 0.15 mol l^−1^. The starting systems were assembled with the input generator CHARMM-GUI.^48^

All the MD simulations were performed with the program packages Amber20 and AmberTools21.^49^**[**1**]**^**2+**^ was described using the generalized Amber force field included in AmberTools21, with ground state point charges computed within the restrained electrostatic potential atomic partial charge (RESP) scheme in Gaussian16 on the RB3LYP/def2-SVP level of theory (see above). The lipids were described using the Lipid17 force field, while water and the atomic ions were described using the “optimal” point charge model^50^ implemented in AmberTools21. After a 10 000 step minimization with 5000 steps employing the steepest gradient algorithm and another 5000 steps using a conjugate gradient algorithm, the systems were heated using a Langevin thermostat at a collision frequency of 1.0 ps^−1^ to 100 K in 2500 time steps (5 ps), followed by heating to 300 K in 50 000 steps (100 ps). Finally, the systems were propagated until the membrane was assembled and all water pores had disappeared. The insertion mode of **[**1**]**^**2+**^ was then investigated by eye.

For all simulations, a time step of 2 fs was used with the SHAKE algorithm^51^ applied to freeze hydrogen bonds at a relative geometric tolerance of 1.10^−7^, thus enabling large time steps. Constant pressure periodic boundary conditions were superimposed with anisotropic pressure scaling at a pressure relaxation time of 1 ps, which was increased to 2 ps for the second heating phase only. The cutoff for non-bonded interaction terms was set to 10 Å. The simulations were performed using the GPU (CUDA) version of pmemd.^52–54^

In general, simulations with fewer (150) DOPG molecules ran more stably and were more likely to assemble a membrane. By contrast, the higher number of DOPG molecules (170) led to more simulations crashing due to distortion of the rectangular periodic box and lipid molecules clump together more often, not forming a membrane. Additionally, the CHARMM-GUI input generator with which we obtained the systems prompted a warning in the 170 DOPG simulations, claiming that too little volume of solvent is left after the placement of **[**1**]**^**2+**^ and the lipid molecules. As a result, more trajectories with 170 DOPG molecules needed to be executed to compensate for the crashing trajectories. This also allowed us to obtain more solid statistics in the latter case, as here the difference in parallel and transmembrane insertion modes was smaller compared to the 150 DOPG simulations.

## Conflicts of interest

There are no conflicts to declare.

## Supplementary Material

ME-008-D2ME00243D-s001
