# Supplementary material for: Alignment and photooxidation dynamics of a perylene diimide chromophore in lipid bilayers
Source: Mol Syst Des Eng. 2023 Feb 22;8(7):842–52. doi: 10.1039/d2me00243d (PMC10317050; doi:10.1039/d2me00243d)
Supplement: ME-008-D2ME00243D-s001 [file ME-008-D2ME00243D-s001.pdf]

Electronic Supplementary Material (ESI) for Molecular Systems Design & Engineering.
This journal is © The Royal Society of Chemistry 2023

Supporting Information

Alignment and photooxidation dynamics of a perylene diimide chromophore in lipid bilayers

Authors:

Novitasari Sinambela,[a] Richard Jacobi,[b,c] David Hernández-Castillo,[b,c] Elisabeth Hofmeister,[f] Nina Hagmeyer,[e,f] Benjamin Dietzek-Ivanšić,[e,f] Leticia González,[b,d] and Andrea Pannwitz[a]

[a]Institute of Inorganic Chemistry I, Ulm University, Albert-Einstein-Allee 11, 89081 Ulm, Germany

[b]Institute of Theoretical Chemistry, Faculty of Chemistry, University of Vienna, Währinger Straße 17, 1090 Vienna, Austria

[c]Doctoral School in Chemistry (DoSChem), University of Vienna, Währinger Straße 42, 1090 Vienna, Austria

[d]Vienna Research Platform on Accelerating Photoreaction Discovery, University of Vienna, Vienna, Austria, Währinger Straße 17, 1090 Vienna

[e]Institute of Physical Chemistry and Abbe Center of Photonics, Friedrich Schiller University Jena, Helmholtzweg 4, Jena 07743, Germany

[f]Leibniz Institute of Photonic Technology (IPHT), Research Department Functional Interfaces, Albert-Einstein-Straβe 9, Jena 07745, Germany

Contents

S1. Synthesis and characterization of 2 and $[1]^{2+}$ 3

Synthesis of N,N'-di(butylenedimethylamine)-3,4,9,10-perylenediimide (2) 3

Synthesis of N,N'-di(butylenetrimethylammonium)-3,4,9,10-perylenediimide $[1]^{2+}$ as PF_6 salt 4

S2. Photooxidation of $[1]^{2+}$ and temporally resolved UV-vis spectra in DOPG 6

S3. Stern-Volmer quenching experiment 7

S4. Typical hydrodynamic diameter of DOPG liposome containing $[1]^{2+}$ 11

S5. Coordinates of $[1]^{2+}$ 11

References 14

S1. Synthesis and characterization of 2 and $[1]^{2+}$

Synthesis of N,N'-di(butylenedimethylamine)-3,4,9,10-perylenediimide (2)

In 20 mL of isobutanol perylene tetracarboxylic dianhydride (0.100 g, 0.255 mmol, 1.00 eq.) and (4-aminobutyl) dimethylamine (0.14 mL, 1.02 mmol, 4.00 eq.) were combined, stirred and heated at 90 °C for 24 h under argon atmosphere. After the mixture was cooled to room temperature, the crude product was collected by filtration. To remove unreacted PTCDA, to the mixture was added 5% aqueous NaOH solution and stirred at 90 °C for 30 minutes. The product was separated from solvent by filtration and washed with water and ethanol. The red solid powder then dried under vacuum to give 0.14 g (0.238 mmol, 95% yield) product.

^{1}H NMR (400 MHz, $CDCl_3$) δ 8.61 (d, *J* = 8.0 Hz, 4H), 8.53 (d, *J* = 8.1 Hz, 4H), 4.20 (t, 4H), 2.45 – 2.23 (m, 4H), 2.22 (s, 12H), 1.86 – 1.67 (m, 4H), 1.61 – 1.39 (m, 4H).

MALDI-MS: calcd. for $[C_{36}H_{36}N_4O_4]^+$ (m/z): 588.27, found: 588.27

Anal. calcd. for $C_{36}H_{36}N_4O_4$: C, 73.45; H, 6.16; N, 9.52; O, 10.87, found C, 73.16; H, 6.15; N, 9.49; O, 10.96.

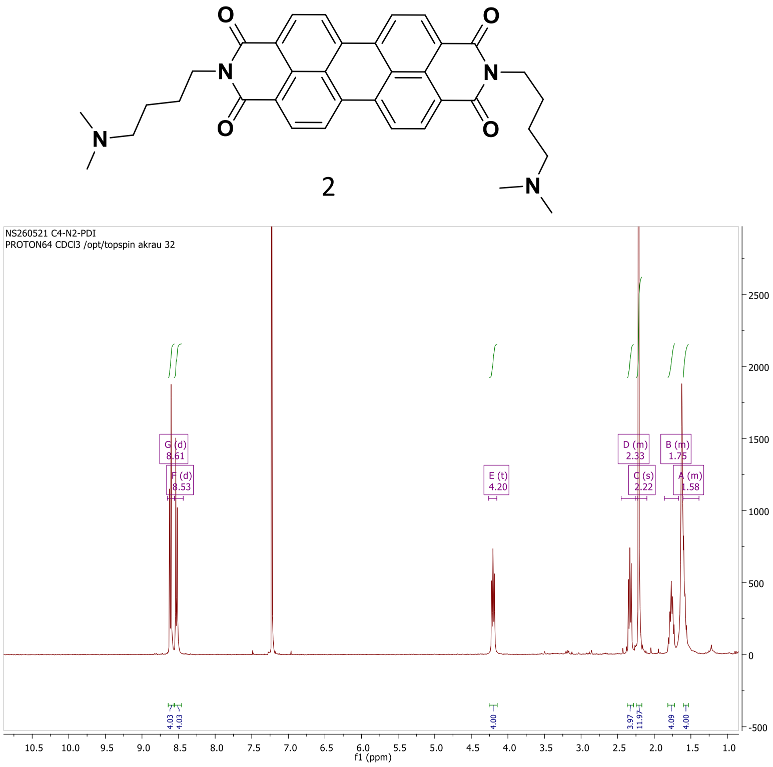

Figure S1.1: ^{1}H NMR spectrum of 2 in $CDCl_3$.

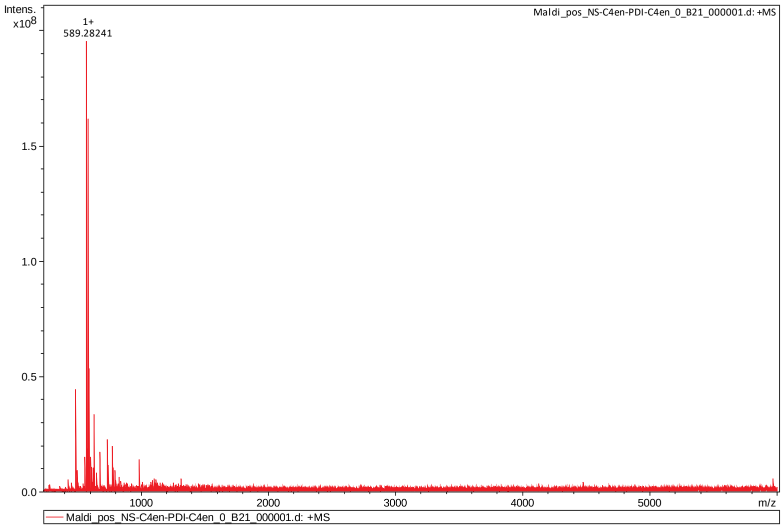

Figure S1.2: mass spectrum of 2.

Synthesis of N,N'-di(butylenetrimethylammonium)-3,4,9,10-perylenediimide $[1]^{2+}$ as PF_6 salt

A degassed solution of N,N'-di(butylenedimethylamine)-3,4,9,10-perylenediimide (0.100 g, 0.170 mmol, 1 eq.) and methyl iodide (0.287 mL, 1.70 mmol, 10 eq.) in 10 mL of toluene was refluxed for 3 h under argon atmosphere. The suspended mixture was collected by filtration, washed with ether and dried under vacuum. To the aqueous solution of the obtained compound was added dropwise to an aqueous solution of NH_4PF_6. After stirring of the solution at 65 °C for 1 h, the precipitate was centrifugated, washed with water to remove produced exchanged salts and dried under vacuum to give 0.12 g (0.130 mmol, 76 % yield) of the desired product.

^{1}H NMR (400 MHz, CD_3CN) δ 8.47 – 8.30 (m, 8H), 4.18 (t, *J* = 6.7 Hz, 4H), 3.39 – 3.29 (m, 4H), 3.04 (s, 18H), 1.85 – 1.70 (m, 8H).

MALDI-MS: calcd. for $[C_{38}H_{42}F_6N_4O_4P]^{2+}$(m/z): 763.28, found: 763.28

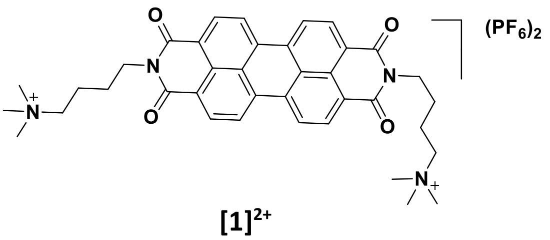

$[1]^{2+}$

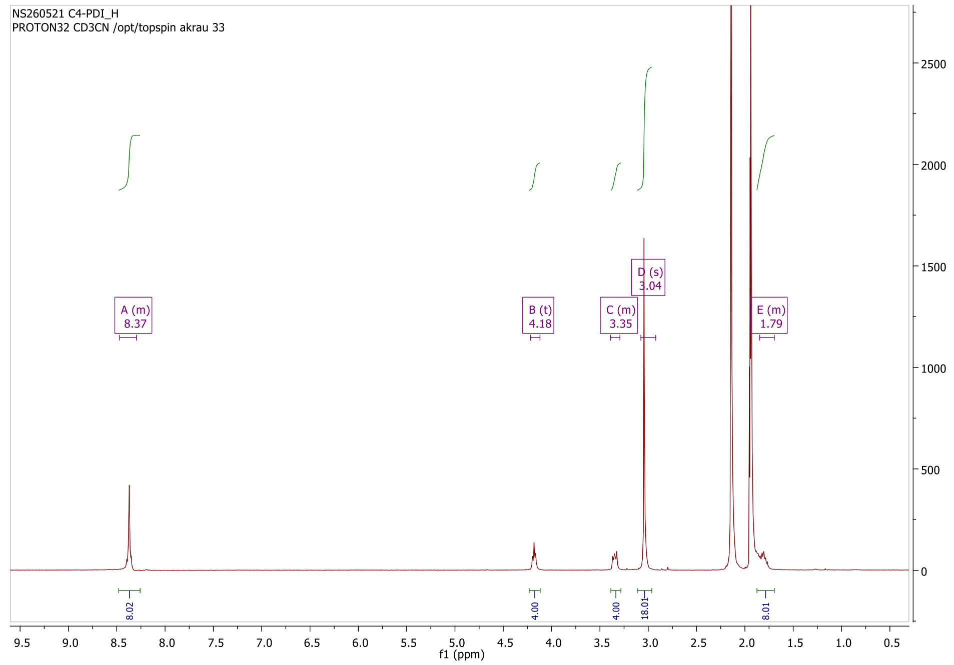

Figure S1.3: ^{1}H NMR spectrum of **[1]$^{2+}$**.

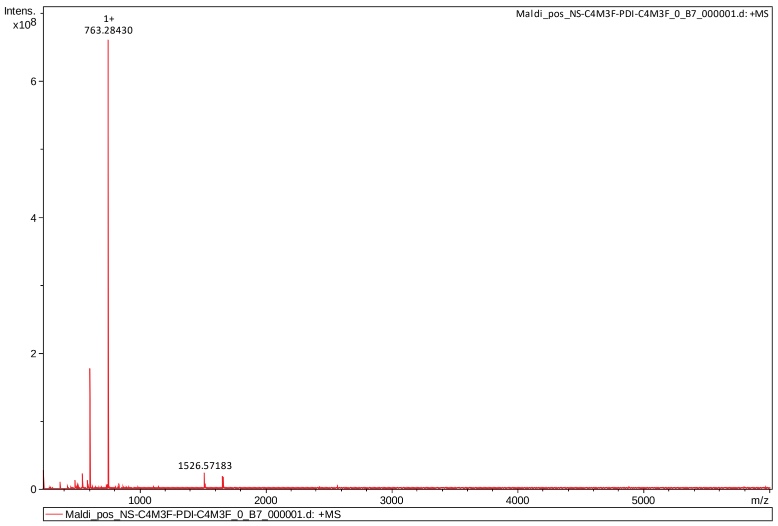

Figure S1.4: mass spectrum of **[1]$^{2+}$**.

Anal. calcd. for $C_{38}H_{42}F_{12}N_4O_4P_2 \cdot 0.1\ NH_4PF_6$: C, 49.34; H, 4.62; N, 6.21; found C, 49.53; H, 4.81; N, 6.07.

S2. Photooxidation of [1]$^{2+}$ and temporally resolved UV-vis spectra in DOPG

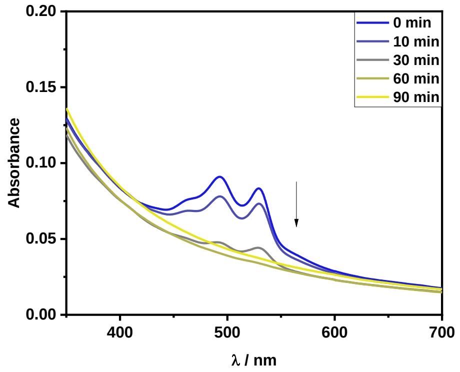

Figure S2.1. Photooxidation of **[1]**$^{2+}$ and temporally resolved UV-vis spectra in DOPG:(14:0 PEG2000 PE):**[1]**$^{2+}$ = 100:1:1 liposomes in phosphate buffer (10 mM, pH 7.0) without size exclusion.

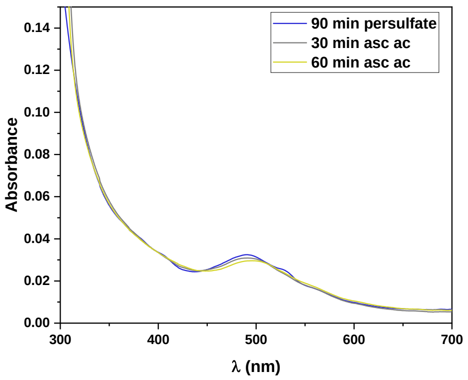

Figure S2.2. UV-vis spectra of Addition of photooxidized **[1]**$^{2+}$ upon addition of 100 mM of ascorbic acid. Experimental conditions: DOPG:(14:0 PEG2000 PE):**[1]**$^{2+}$ = 100:1:1 liposomes in phosphate buffer (10 mM, pH 7.0) without size exclusion with 50 mM of sodium persulfate after irradiation of 90 min and afterward additional 100 mM of ascorbic acid.

S3. Stern-Volmer quenching experiment

Solutions were prepared as described in main pages.

In cuvette:

V = 3 mL

[1]$^{2+}$ = 1 uM

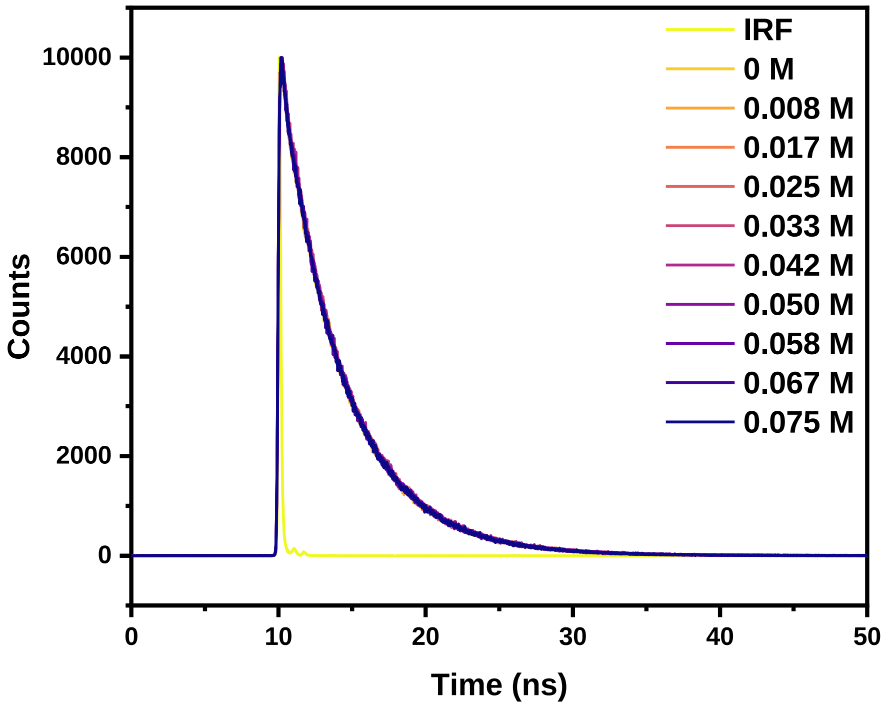

Figure S3.1. Kinetic traces of luminescence decay upon excitation at 450 nm in acetonitrile/water 1:1 (V/V) and 1 μM **[1]**$^{2+}$ and various concentrations of $Na_2S_2O_8$ as quencher.

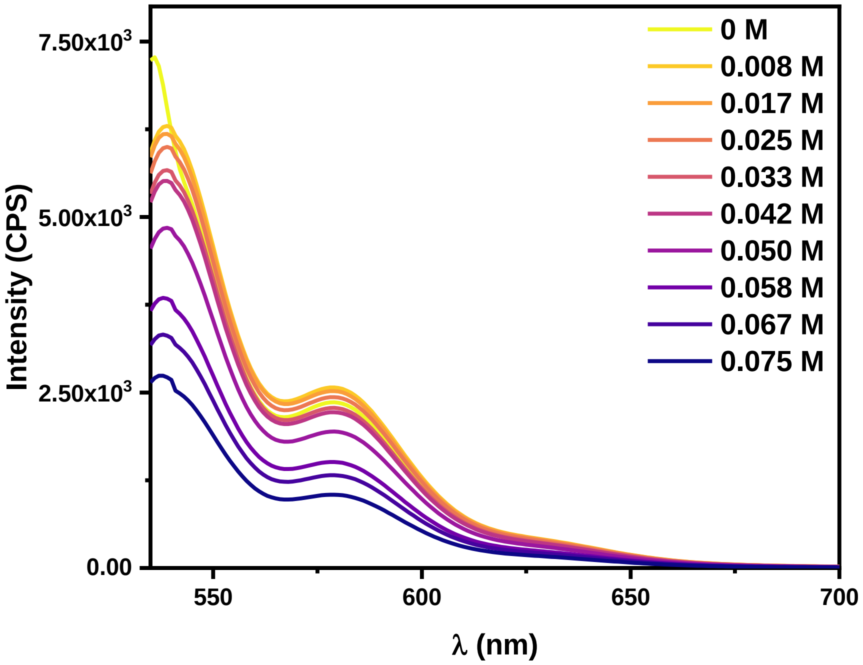

Figure S3.2. Luminescence quenching upon addition various concentrations of $Na_2S_2O_8$ in acetonitrile/water 1:1 (V/V) and 1 μM $\mathbf{[1]}^{2+}$.

Liposomes were prepared as described in methods section.

In cuvette:

V = 3 mL

$\mathbf{[1]}^{2+}$= 1.67 μM

[DOPG] = 167 μM

[14:0 PEG2000 PE] = 0.17 μM

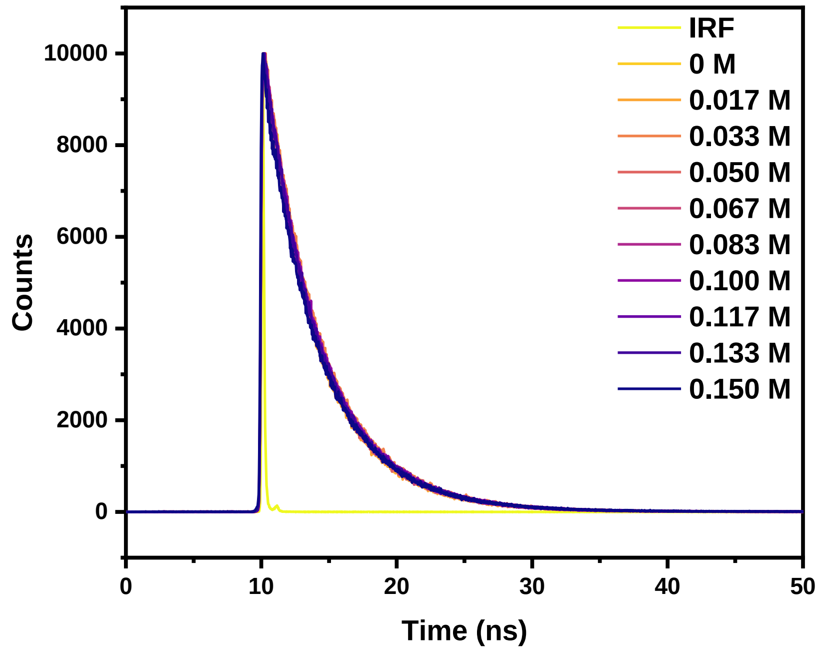

Figure S3.3. Kinetic traces of luminescence decay upon excitation at 450 nm at pH 7.0 of DOPG liposomes without size exclusion chromatography with 1% (14:0 PEG2000 PE), 1% **[1]**$^{2+}$ and various concentrations of $Na_2S_2O_8$ as quencher.

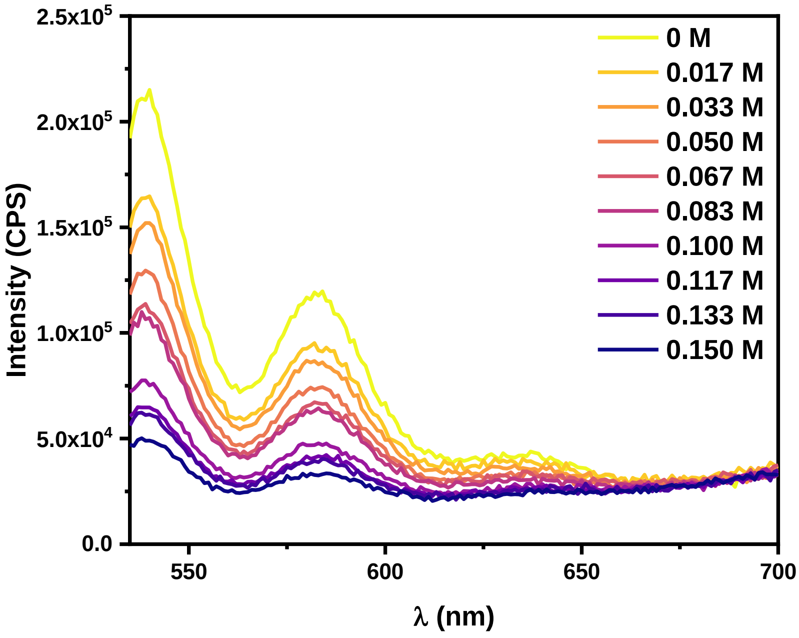

Figure S3.4. Luminescence quenching upon addition various concentrations of $Na_2S_2O_8$ in DOPG liposomes without size exclusion chromatography with 1% (14:0 PEG2000 PE), 1% **[1]**$^{2+}$.

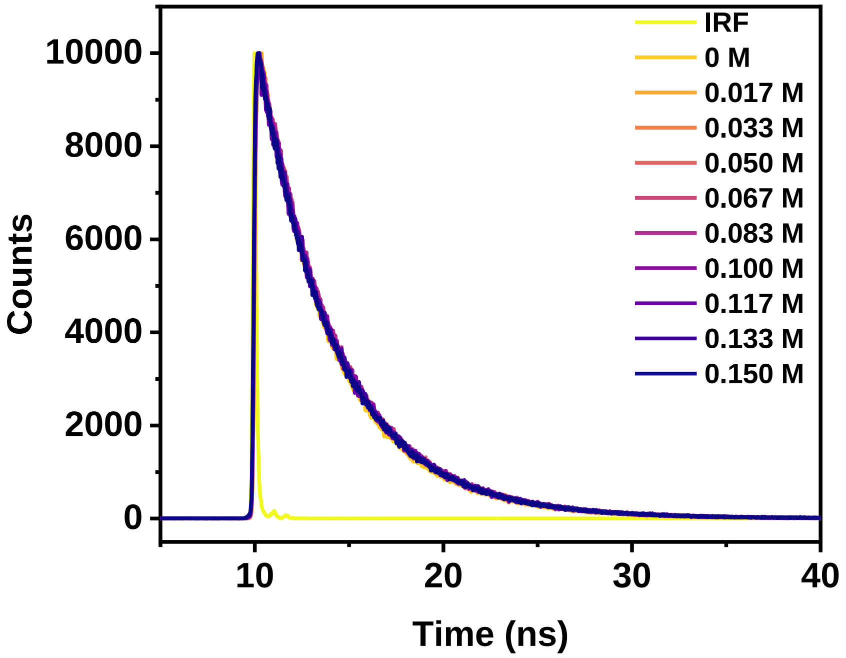

Figure S3.5. Kinetic traces of luminescence decay upon excitation at 450 nm at pH 7.0 of DOPG liposomes with size exclusion chromatography with 1% (14:0 PEG2000 PE), 1% **[1]**$^{2+}$ and various concentrations of $Na_2S_2O_8$ as quencher.

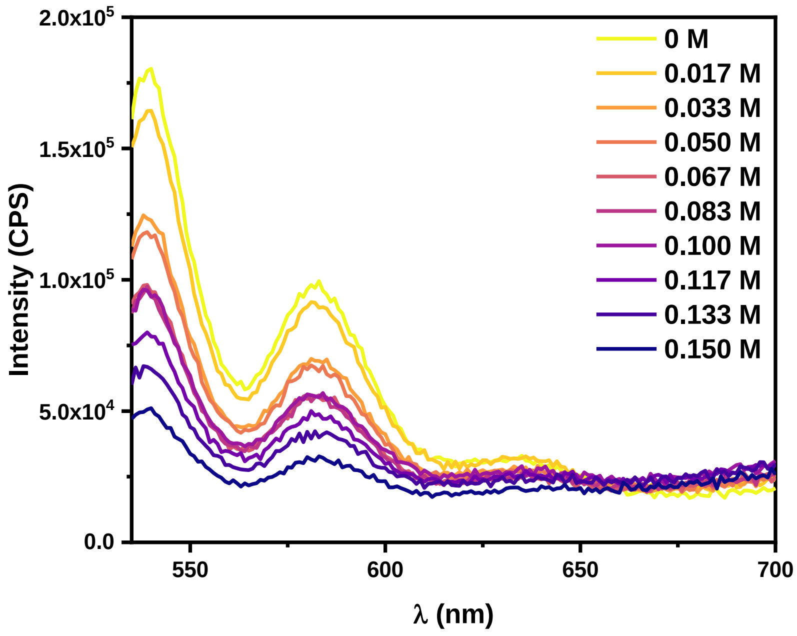

Figure S3.6. Luminescence quenching upon addition various concentrations of $Na_2S_2O_8$ in DOPG liposomes with size exclusion chromatography with 1% (14:0 PEG2000 PE), 1% **[1]**$^{2+}$.

S4. Typical hydrodynamic diameter of DOPG liposome containing $[1]^{2+}$

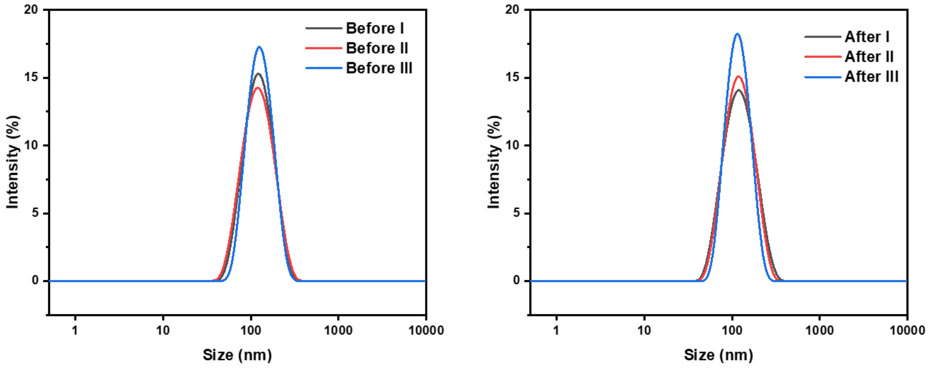

Figure S4.1. DLS measurements of DOPG liposomes with size exclusion chromatography with 1% (14:0 PEG2000 PE), 1% $\mathbf{[1]^{2+}}$**,** before (left) and after (right) photooxidation reaction.

Table S1. DLS data: average size and polydispersity index, before and after photooxidation reaction.

Parameters	Before	After
	Mean	Mean
Z-average (nm)	117	111
Polydispersity index	0.11	0.07

S5. Coordinates of $[1]^{2+}$

88

N-substituted perylene diimide

C	-2.87525	0.33453	-2.20917
C	-1.47598	0.33312	-2.22232
C	-0.73452	-0.46864	-1.34924
C	-1.43165	-1.31061	-0.42502
C	-2.86098	-1.30408	-0.41931
C	-3.57197	-0.46982	-1.31791
C	0.73451	-0.46862	-1.34924
C	-0.73462	-2.15708	0.49533
C	0.73464	-2.15708	0.49532
C	1.43166	-1.31061	-0.42504
C	1.47747	-2.95501	1.37056

C	-1.47744	-2.95499	1.37060
C	-2.87715	-2.94018	1.37093
C	-3.57316	-2.12443	0.49022
H	-3.43712	-3.56719	2.06556
H	-3.43370	0.96855	-2.89845
C	1.47596	0.33316	-2.22231
C	2.87523	0.33458	-2.20917
C	3.57196	-0.46978	-1.31792
C	2.86099	-1.30406	-0.41934
H	3.43368	0.96862	-2.89844
C	2.87718	-2.94020	1.37088
H	3.43716	-3.56722	2.06548
C	3.57318	-2.12442	0.49017
H	-0.97164	0.98102	-2.93676
H	0.97162	0.98107	-2.93673
H	0.97373	-3.60899	2.08003
H	-0.97368	-3.60896	2.08008
C	-5.04956	-0.43312	-1.30725
C	-5.05192	-2.11493	0.51782
N	-5.69935	-1.25468	-0.38311
O	-5.69000	0.31268	-2.03849
O	-5.70382	-2.80106	1.28843
C	5.04955	-0.43307	-1.30728
C	5.05195	-2.11492	0.51775
N	5.69936	-1.25465	-0.38317
O	5.68999	0.31274	-2.03851
O	5.70385	-2.80107	1.28834
C	7.17126	-1.23607	-0.34782
H	7.49511	-2.26562	-0.15655
H	7.50873	-0.93520	-1.34588
C	7.77571	-0.31772	0.71914

H	8.86068	-0.50404	0.70476
H	7.42635	-0.63122	1.71627
C	7.54817	1.19116	0.51366
H	8.35409	1.74123	1.02110
H	7.62013	1.41482	-0.55943
C	6.20222	1.63437	1.07170
H	6.18316	1.52365	2.16409
H	5.38163	1.03334	0.67076
C	4.40618	3.26992	1.28716
C	6.71981	4.02413	1.51548
C	5.84735	3.37232	-0.68138
N	5.81032	3.07731	0.79141
C	-7.17125	-1.23611	-0.34775
H	-7.49509	-2.26566	-0.15645
H	-7.50873	-0.93526	-1.34581
C	-7.77569	-0.31774	0.71921
H	-8.86066	-0.50407	0.70485
H	-7.42630	-0.63121	1.71633
C	-7.54817	1.19114	0.51368
H	-8.35408	1.74122	1.02113
H	-7.62017	1.41477	-0.55941
C	-6.20221	1.63439	1.07167
H	-6.18312	1.52370	2.16406
H	-5.38162	1.03336	0.67072
C	-6.71983	4.02415	1.51539
C	-4.40620	3.26999	1.28702
C	-5.84743	3.37229	-0.68148
N	-5.81034	3.07733	0.79132
H	3.73695	2.62275	0.70905
H	4.12458	4.31999	1.15234
H	4.36511	3.00154	2.34872

H	7.74546	3.88133	1.16150
H	6.65893	3.82105	2.59060
H	6.39493	5.04953	1.30641
H	6.88824	3.40953	-1.01599
H	5.37154	4.34450	-0.85021
H	5.31473	2.57963	-1.21833
H	-6.39497	5.04955	1.30629
H	-6.65892	3.82110	2.59052
H	-7.74549	3.88133	1.16144
H	-4.36509	3.00163	2.34859
H	-4.12463	4.32006	1.15217
H	-3.73697	2.62281	0.70891
H	-5.31481	2.57960	-1.21841
H	-5.37164	4.34448	-0.85035
H	-6.88832	3.40948	-1.01606

References

[1] B. Limburg, E. Bouwman, S. Bonnet, *Chem. Commun* **2015**, *51*, 17128.
